# Odor prediction of whiskies based on their molecular composition

**DOI:** 10.1038/s42004-024-01373-2

**Published:** 2024-12-19

**Authors:** Satnam Singh, Doris Schicker, Helen Haug, Tilman Sauerwald, Andreas T. Grasskamp

**Affiliations:** 1 Department of Sensory Analytics and Technologies, Fraunhofer Institute for Process Engineering and Packaging IVV, Freising, Germany; 2 Department of Psychiatry and Psychotherapy, Friedrich-Alexander-Universität Erlangen-Nürnberg, Erlangen, Germany; 3 Department of Chemistry and Pharmacy, Friedrich-Alexander-Universität Erlangen-Nürnberg, Erlangen, Germany; 4 Department of Systems Engineering, Saarland University, Saarbrücken, Germany

**Keywords:** Cheminformatics, Mass spectrometry

## Abstract

Aroma compositions are usually complex mixtures of odor-active compounds exhibiting diverse molecular structures. Due to chemical interactions of these compounds in the olfactory system, assessing or even predicting the olfactory quality of such mixtures is a difficult task, not only for statistical models, but even for trained assessors. Here, we combine fast automated analytical assessment tools with human sensory data of 11 experienced panelists and machine learning algorithms. Using 16 previously analyzed whisky samples (American or Scotch origin), we apply the linear classifier OWSum to distinguish the samples based on their detected molecules and to gain insights into the key molecular structure characteristics and odor descriptors for sample type. Moreover, we use OWSum and a Convolutional Neural Network (CNN) architecture to classify the five most relevant odor attributes of each sample and predict their sensory scores with promising accuracies (up to F1: 0.71, MCC: 0.68, ROCAUC: 0.78). The predictions outperform the inter-panelist agreement and thus demonstrate previously impossible data-driven sensory assessment in mixtures.

## Abstract (web summary)

Aroma compositions are usually complex mixtures of odor-active compounds exhibiting diverse molecular structures, and assessing or predicting the olfactory qualities of such mixtures is challenging. Here, fast automated analytical assessment tools are combined with the human sensory data of 11 experienced panelists and machine learning algorithms, enabling samples to be distinguished and classified based on their detected molecules, and gaining insights into key molecular structure characteristics and odor descriptors.

## Introduction

Odors are ubiquitous in our environment and are perceived either consciously or in the background. Most of these odors are a complex mixture of diverse odor molecules, creating a specific odor impression. While some characteristic odors are mainly determined by single molecules, e.g., vanillin^1^, most food aromas consist of a whole range of molecules. One prominent example is the whisky spirit, whose aroma profile can be determined from more than 40 compounds^2^ and which can consist of even more non-odorous volatiles^3^. As diverse as these molecules are the aroma impressions and, by proxy, the odor descriptors that best describe the resulting aroma^4^. Rapid sensory evaluation methods as well as analytical methods for analyte detection allow a distinction between specific types of whiskies^5^. This shows that aroma evaluation can also be used to investigate further aspects of food products apart from smell perception.

Human panels are widely used to evaluate flavors. However, as olfactory perception is rather subjective and acquiring comprehensive quantifiable measurements is difficult, it is important to use odor descriptors based on the evaluations of multiple subjects for a consensus. Moreover, other senses, experiences, personality, and biological circumstances can also influence the final perception of a participant^6–9^. Overall, comparability between participants when describing odors is limited, no matter if they are experts or novices^10^, increasing the difficulty to classify odors due to ambiguity in the chosen descriptors. However, panelist training can enhance identification performance, consensus, and terminology^11,12^.

Despite difficulties, rapid sensory evaluation methods like *r*ate-*a*ll-*t*hat-*a*pply (RATA)^13^ can be effective, but these methods still require an immense amount of invested time, money, and often trained panelists. Alternatively, *m*achine *l*earning (ML) methods have the potential to amend the knowledge of panelists and could be used to predict the odor of molecules quickly, accurately, and reliably, thus reducing the overall time and effort required. ML methods could thus support and supplement human sensory evaluation, for example by pre-selecting promising odorants.

In the last few years, enormous progress has been made in automated odor prediction^14–23^. The most widely utilized methods vary between different machine-learning algorithms like decision trees, *R*andom *F*orests (RF), graph-based approaches, and linear methods. Additionally, different features are used as inputs, ranging from mass spectra over physicochemical properties to solely structural characteristics described using textual inputs such as SMILES^24^. Although ML methods cannot replace human panels so far, molecular odor prediction can already reach human-level performance for specific descriptors^22^. The aforementioned methods mainly focus on individual odorant molecules. However, as discussed above, everyday odors are seldom monomolecular but rather a mixture of diverse odorants. Thus, in addition to the complexity of structure-odor relationships^25^, interaction effects also occur between the different odorants. This is demonstrated by the limited ability of humans to identify odorants in ternary or higher mixtures^26,27^ even after extensive training^28^.

For classification of odors, analytical devices such as *e*lectronic noses (e-noses), or mass spectrometric methods can be applied^14,29^. Regarding odor mixtures, on the one hand, previous work predicted the responses of e-noses based on the known responses to their individual components^30,31^ as well as the mass spectra from the odor impressions of mixtures^32^. On the other hand, e-noses, combined with artificial neural networks, were shown to help monitoring environmental odors—consisting of a multitude of odorants—regarding concentrations and odor classes^33^.

Prediction of human odor perception per se has already targeted aroma mixtures regarding intensity^34–36^, pleasantness^37^, and sweetness impression^38^. Further, previous work has used mass spectra of essential oils to predict their odor impression^39^. Therefore, several odor descriptors were combined in five odor descriptor groups, with the method achieving a true positive prediction accuracy of ~70% and a true negative prediction of ~50%. Although this is a promising step, there is still room for improvement. Moreover, in their approach, the authors combine descriptors using pretrained vectors based on English Wikipedia and Fast Text^40–42^. However, as discussed in Sisson^43^, usage of descriptor words might vary significantly in their common usage and in olfactory context leading to creation of embedding vectors that carry a different context than intended. Odor quality in the context of odor mixtures is thus still a challenge and more research is needed to test different approaches.

Moreover, to not only be useful in research aspects and theory but also in practice, the whole process for odorant prediction should not only target odor mixtures, but also keep in mind a fast and easy data generation to be used as input. This starts with the analysis of an odor, e.g., by chemically analyzing and decomposing a mixture into its single aroma compounds with *g*as *c*hromatography–*m*ass *s*pectrometry (GC–MS). This can be coupled with automatic molecule detection. Within this study, we considered this whole pipeline by combining data of previously published studies. Thus, the aim of our work is to extend the current singular structure-odor prediction models to molecular mixtures, which is closer to real-world applications.

As such, like in our previous work, we used molecular mixtures from American and Scotch whisky samples that were determined by GC–MS coupled with automatic compound detection analysis^5,44^. Further, we used sensory data, generated by a human expert panel using RATA^5^, to determine the top-5 odor descriptors per whisky.

For prediction, we used two algorithms: On one hand, we applied the comprehensive linear model OWSum (*O*lfactory *W*eighted *S*um) that provides insight into the classification decision process^21^. Using OWSum, we firstly investigated whether the type of whisky can be correctly predicted based on the detected molecules or the top-5 odor descriptors. This enables us to also get insight into the molecular and sensory distinguishability of American and Scotch whisky and the impact of olfactory and molecular features. Secondly, we used OWSum to predict the top-5 odor descriptors based on the detected molecules in each sample. On the other hand, we used a *C*onvolutional *N*eural *N*etwork (CNN) architecture to predict the odor qualities of the whiskies based on the substructural similarity features of these detected molecules. We compared our odor prediction results with inter-subject reliability to estimate their performance as well as against educated guessing, i.e., guessing the most frequently occurring descriptors in the dataset and also against two benchmarking methods, i.e., linear *S*upport *V*ector *M*achines (SVM) and RF.

## Results

### Predicting the type of whisky with OWSum

First, we explored the capability of the linear classification algorithm OWSum using different weighting schemes to accurately distinguish whiskies into American or Scotch based on either panel descriptors or detected molecules. When top-5 odor descriptors were used as features, the same-weighted CP1 variant worked the best with 93.75% accuracy for *l*eave-*o*ne-*o*ut validation (LOO). When molecules were used as features, applying *t*erm *f*requency-*i*nverse *d*ocument *f*requency (tf-idf) weights worked better than the same-weighted variant and achieved 100% accuracy for CP1 and CP2 (see Supplementary Material Table S1 for the performance of all tested variations). Overall, OWSum could reliably predict the type of whisky in both cases (see Table 1).

**Table 1:** Accuracy for LOO to predict the type of whisky (American or Scotch) using OWSum

Model	Features	ACC [%]
OWSum (CP1, same-weighted)	Descriptors	93.75
OWSum (CP2, same-weighted)	Descriptors	81.25
OWSum (CP1, tf-idf-weighted)	Molecules	100
OWSum (CP2, tf-idf-weighted)	Molecules	100

**Fig. 1:**
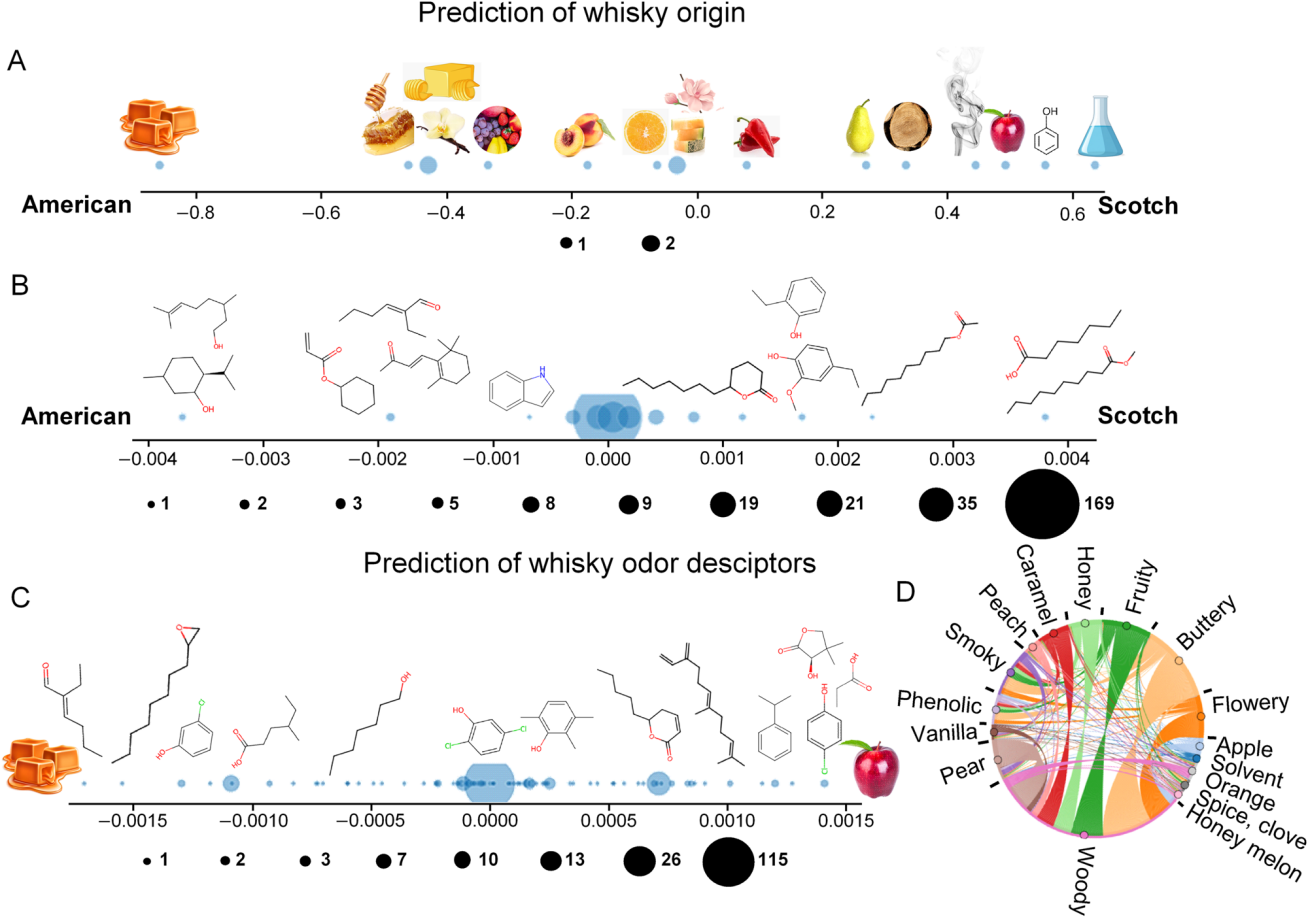
Insight into feature-class relationships using OWSum. The x-axis values represent the differences between the influence values of the two respective classes. **A** Prediction of the whisky type (American vs. Scotch) based on descriptors with same-weighted CP1 OWSum, re-creation accuracy: 93.75%. **B** Prediction of the whisky type based on molecules with tf-idf-weighted CP1 OWSum, re-creation accuracy: 100%. **C** Prediction of the odor descriptors of a whisky based on molecules with tf-idf-weighted CP2 OWSum, re-creation accuracy: 96.88%. We show the importance of features for “caramel” vs. “apple”. **D** Bokeh-diagram of the dissimilarity between descriptors, the arc width displays the pairwise dissimilarity by summing all influence value differences per class (for better visualizing arc width = 1.1^abs(“sum of influence values differences” × 1000)). Dots represent the number of respective descriptors (for **A**) or molecules (for **B**, **C**). We depict some of the molecules as examples. This image was created with resources from Freepik.com.

Similar to other explainability methods such as calculation of feature importance values, the nature of OWSum allows gaining insight into the classification and thus identify the most important features. Therefore, in a second analysis, we trained on all detected molecules to gain as much information as possible for identifying characteristic molecules and structural patterns corresponding to samples belonging in each of the two classes. To get a measure of how valid this information is, we predicted for each whisky its type based on this model and thus “re-created” the type of whisky based on the features. By this, we got a re-creation accuracy, i.e., the comparison of the true with the predicted type if using the same train and test data, of 100% and 93.75% respectively that justified the validity of the insight. *Caramel-like* was identified as the most characteristic odor descriptor for American whereas *apple-like*, *phenolic*, and *solvent*-*like* odors were more pronounced in Scotch whiskies (Fig. 1A). These results show that there are clear relationships between volatile molecules as well as olfaction with the type of whisky. Mostly the molecules menthol and citronellol pushed the classification towards American whereas methyl decanoate and heptanoic acid had higher impact to classify a whisky as Scotch. Indeed, these four molecules were always present in one class, but never present in the other class, in accordance with our previous work on this dataset that identified these molecules to be exclusive for American or Scotch whiskies^5^. However, this accounts for our limited dataset and does not necessarily represent all Scotch and American whiskies or an exhaustive list of all molecules found in these whiskies. Though applying the method in real-world thus could lead to lower accuracies, it still confirms the discriminative power of odorants to distinguish between whisky types^3^. Moreover, OWSum can be used to assign numerical values for all molecules, which illustrates their predictive power for classifying the whisky as American or Scotch (see Fig. 1B). Our results show that there are clear relationships between volatile molecules as well as olfaction with the type of whisky.

### Predicting the odor quality of whiskies with OWSum and CNN

**Fig. 2:**
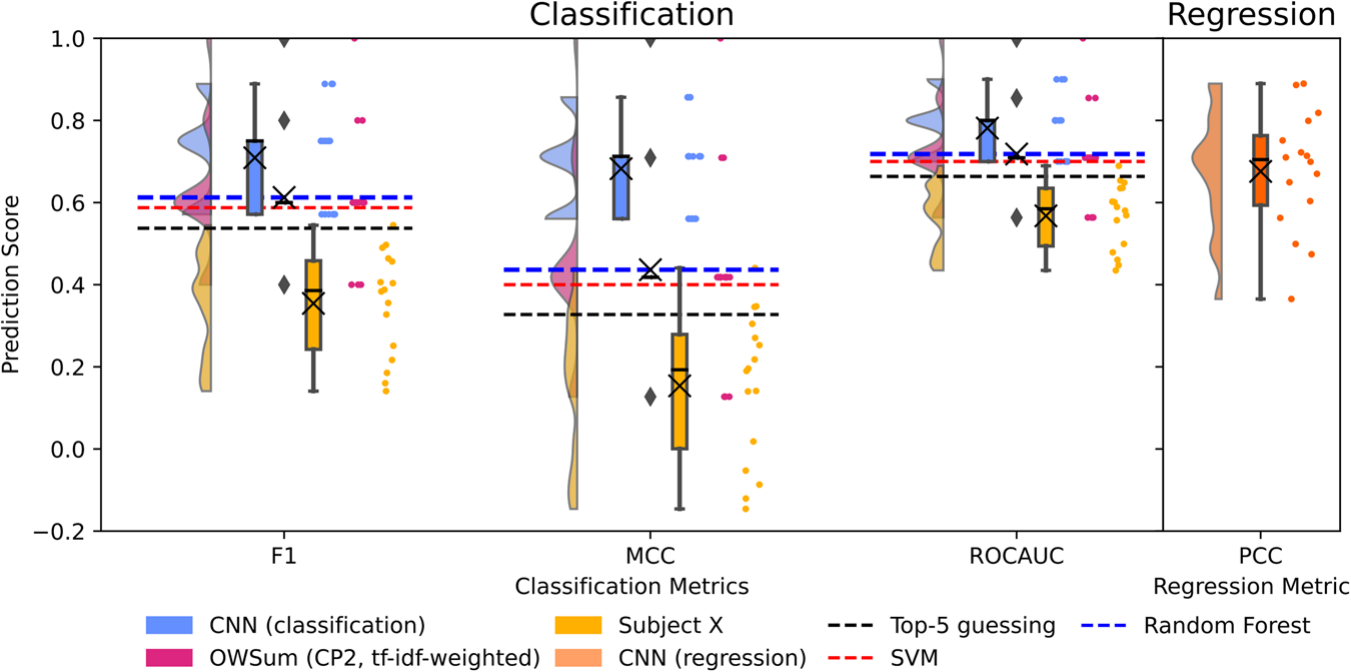
Insight into the evaluation metrics. Insight into the evaluation metrics using all 16 LOO iterations for CNN pipeline (shown in blue for classification and orange for regression), OWSum (pink) and Subject X (yellow) results using a raincloud plot (code based on ptitprince 0.2.7^56^). For CNN and OWSum, dots represent the respective evaluation metric per LOO iteration and as such per whisky. For Subject X, dots represent the respective evaluation metric per subject, aggregated over all whiskies. Clouds illustrate the data distribution. Crosses in the boxplots depict the mean value of the respective metric, solid black lines within the boxplots the median. Black dashed lines show the metrics for educated guessing, i.e., if the five most occurring descriptors of all but one whisky are predicted for the omitted whisky. Blue dashed lines show the metrics for RF and red dashed lines for SVM. See Table S2 for statistical details. F1 micro F1-Score, ROCAUC *A*rea *U*nder the *R*eceiver *O*perating *C*haracteristic *C*urve, MCC micro *M*atthews *C*orrelation *C*oefficient, PCC *P*earson *C*orrelation *C*oefficient.

In a next step, we wanted to analyze whether the smell of a whisky, represented by the top-5 odor descriptors, can be predicted using either OWSum based on molecules or a CNN based on structural patterns. The results are summarized in Table 2 as well as Fig. 2. Here, 'Subject X' denotes the average inter-subject performance calculated by treating Subject X as the prediction and all subjects but X as the ground truth. The performance metrics were calculated for each of the panelists as Subject X and averaged out. This allows us to set an inter-panelist baseline performance. Table 2 shows that both OWSum and CNN performed better than Subject X, with the CNN pipeline outperforming OWSum. Moreover, both algorithms outperformed the educated top-5 guessing, i.e., if the five most frequent descriptors in the dataset are always chosen as the predictions. We also wanted to analyze how each individual panelist performs compared to the two methods, and if there is any one particular participant that matches or outperforms the algorithms but did not find any. The results are shown in Supplementary Fig. S1.

**Table 2:** Performance to predict the top-5 odor descriptors using OWSum and CNN, as well as inter-subject performance (Subject X) and educated top-5 guessing

Model	F1	MCC	ROCAUC
OWSum (CP1, same-weighted)	0.45	0.20	0.60
OWSum (CP1, tf-idf-weighted)	0.46	0.22	0.61
OWSum (CP2, same-weighted)	0.56	0.38	0.69
OWSum (CP2, tf-idf-weighted)	**0.61**	**0.44**	**0.72**
CNN (with feature scaling)	0.58	0.58	0.71
CNN (without feature scaling)	**0.71**	**0.68**	**0.78**
Subject X	0.35	0.15	0.57
Top-5 guessing	0.52	0.29	0.65
SVM	0.59	0.40	0.70
RF	0.61	0.44	0.72

Rows in bold represent the respective best result for either OWSum or CNN.

While CNN outperforms OWSum in terms of prediction performance, OWSum allows easier insight into the data that is justified by a re-creation accuracy of 96.88% using tf-idf-weights and CP2. As such, molecules can be identified that drive the decision towards a specific descriptor. An example for *apple-like* vs. *caramel-like* is depicted in Fig. 1C. The three molecules with the highest influence value per odor descriptor can be found in Supplementary Material Table S4, however, it should be noted that these influence values should not be interpreted in absolute terms, but rather in relative terms between the classes. Moreover, the aroma of the individual molecules with the highest influence might not be the same as the aroma of the mixture. Further, different descriptors could have a rather similar or distinct shared molecular importance composition. This molecular dissimilarity between descriptors, calculated based on the influence values of OWSum is visualized in Fig. 1D. For example, *butter-like* has a profile quite distinct from *wood-like* (as the arc width is wide), whereas *fruity* and *honey-like* are quite similar.

In addition to odor quality classification, we performed a regression of the RATA scores using CNN. For classification, we found that the CNN with no feature scaling performed slightly better than with scaled features, similar for the regression problem (Table 3).

**Table 3:** Regression performance (PCC) for descriptor ratings using CNN

Model	PCC
CNN (without feature scaling)	0.68
CNN (with feature scaling)	0.47

## Discussion

Even with recent milestone advances in predicting the odor impression of molecules by their structure^21,22^, it has remained notoriously difficult to assess the odor impression of a complex mixture based only on the supposed knowledge of its molecular composition. Besides the known hurdles in identifying individual contained chemicals beyond all reasonable doubt even with state-of-the-art analytical-instrumental solutions, this is mainly due to widely different and often unknown odor thresholds and derived odor activity value of single molecules which also vary between matrices (e.g., air, water, or oil). Therefore, even if all molecules found in a mixture were known, their amounts would not give much of an indication towards their influence on the resulting odor impression. Lastly, even the human nose as a reference will yield results with large inter-subject variability.

Within this work, we investigated the relationship between molecules, odor descriptors, and type of whisky more in detail and predicted the odor of whiskies exemplary for complex odorant mixtures with promising accuracies.

Firstly, we examined the relationships between the two whisky types (American and Scotch) with automatically detected molecules and odor descriptors. Using our own algorithm OWSum^21^ we accurately predicted whether a whisky is American or Scotch based on its molecular composition obtained by efficient analytical assessment of the samples. OWSum not only offers a method to quickly classify whiskies, but also allows us to analyze their ingredients or characteristic features in one step. Making use of this valuable insight into feature-class relationships, we validated our previous work showing that classification of both origins of whisky samples was due to very characteristic components^5,44^. This way, we can also gain certainty that the list of detected molecules is sufficiently meaningful to discriminate between whisky origins. In addition to predict the type of whisky using detected molecules, OWSum achieved high accuracy based on the top-5 odor descriptors, i.e., the five highest descriptors per whisky sample in the dataset. This shows the different and distinguishable sensory profiles of Scotch and American whiskies.

Further, we applied OWSum and CNN to predict the top-5 odor descriptors of the whiskies. It is important to note that, in contrast to the CNN-approach utilized here, we applied OWSum with no structural information for this task, but qualitative lists of molecules. Using high-quality analytical data and modern statistical modeling approaches, we were able to predict the odor impression of complex mixtures with an accuracy that lands within the inter-subject variability. Our algorithmic predictions overall performed even better than the mean trained human subject compared to the rest of the panel as well as better than educated guessing on the top-5 odor descriptors of all-but-one whisky (LOO). As such, in our study we found that educated guesses were more likely to match odor perception of a panel than the rating of an individual subject. Even better, however, are the predictions generated by OWSum and, in particular, CNN. We also trained two different models to use as reference, namely, a RF and a SVM to compare the performances. OWSum performed slightly better than the linear SVM, and just as good as the nonlinear RF. More importantly, the variance between different splits across these methods was higher than OWSum, i.e., OWSum provided more consistent predictions, as shown in Table S2.

None of our input data for the CNN and OWSum contained information about, nor referred directly to, odor activity values or human smell receptor properties. We relied solely on the detected molecules (for OWSum) or the encoding of structural information of each detected molecule (for CNN). Additionally, our CNN-approach worked best when removing information about GC relative peak areas and therefore contained no information about molecule amounts for classification, presumably due to the class weights provided to the *B*inary *C*ross *E*ntropy (BCE) loss function having a stronger influence on the loss function than the scaled features.

Another observation of interest is the variation of the evaluation metrics in each LOO iteration. Using each sample in test and train sets iteratively helps avoid favorable splits along with introduction of class weights, though, the few amounts of data points are still a big constraint for training a model, and availability of more data points would enable a stronger validation of the model. However, these are some of the most decisive factors in this scope of problems. Moreover, this also limits us to evaluate if these results also transfer across different regions of origin and to validate our model on whisky samples from new regions.

Both, the CNN approach and OWSum currently do not consider odor activity values or concentration in making their decisions. Following research should therefore be directed at gaining insight into whether and how odor activity levels or thresholds and odorant-receptor-kinetics can be derived from this or similar approaches. Intuitively, the inclusion of all procurable odor activity levels should lead to even better results. It should also be investigated whether the list of odor descriptors might be further improved to ward off ambiguity^45^ and unify expert assessments as well as account for non-expert sensory data. Finally, as future work, it might be interesting to evaluate the prediction of the aroma of whiskies in a more flexible way, i.e., not considering only the top-5 values but predicting the most applicable probabilities as an aroma profile.

## Methods

### Database

Most data used in this project was taken from previously published works^5,44^. In total, 16 different whiskies, of which nine were Scotch and seven were American whiskies, were previously analyzed using GC–MS and subsequently molecular components automatically matched based on mass spectra and retention indices using an in-house reference library (results previously published in refs. ^5,44^). The analytical data is hence referred to as “molecular composition” within this work. However it has to be noted that this does not cover the exhaustive chemical composition but refers to the detected (volatile) compounds reported in our previous work, as explained in detail in ref. ^5^. Additionally, an expert panel previously evaluated the whiskies’ odor qualities using RATA^13^ on 40% ABV samples as reported in our own work^5^. Each panelist rated the intensities of a maximum of five most applicable attributes out of 17 attributes that were pre-selected by a trained sensory panel. For results and a detailed explanation of these methods, see refs. ^5,44^.

Resulting molecules detected per whisky were used as input for the OWSum algorithm with the aim to predict the type of whisky. Further, substructural patterns were extracted from these molecules and used as inputs for the CNN to predict the odor qualities of the whiskies. These methods are further described here.

### Substructural feature extraction

To extract the substructural features from the detected molecules, we created an applicability matrix that describes the “applicability” or relevance of different substructural patterns that are found across all molecules in our dataset. For this purpose, we first created a reference dataset consisting of 390 commonly found molecules in whisky mixtures from literature^2,3,46–52^. The idea behind using molecules commonly found in whiskies was to extract domain-specific substructural features based on molecules that are expected to be detected using rapid analytical approaches. Thus, allowing this approach to be tuned for different use-cases.

**Fig. 3:**
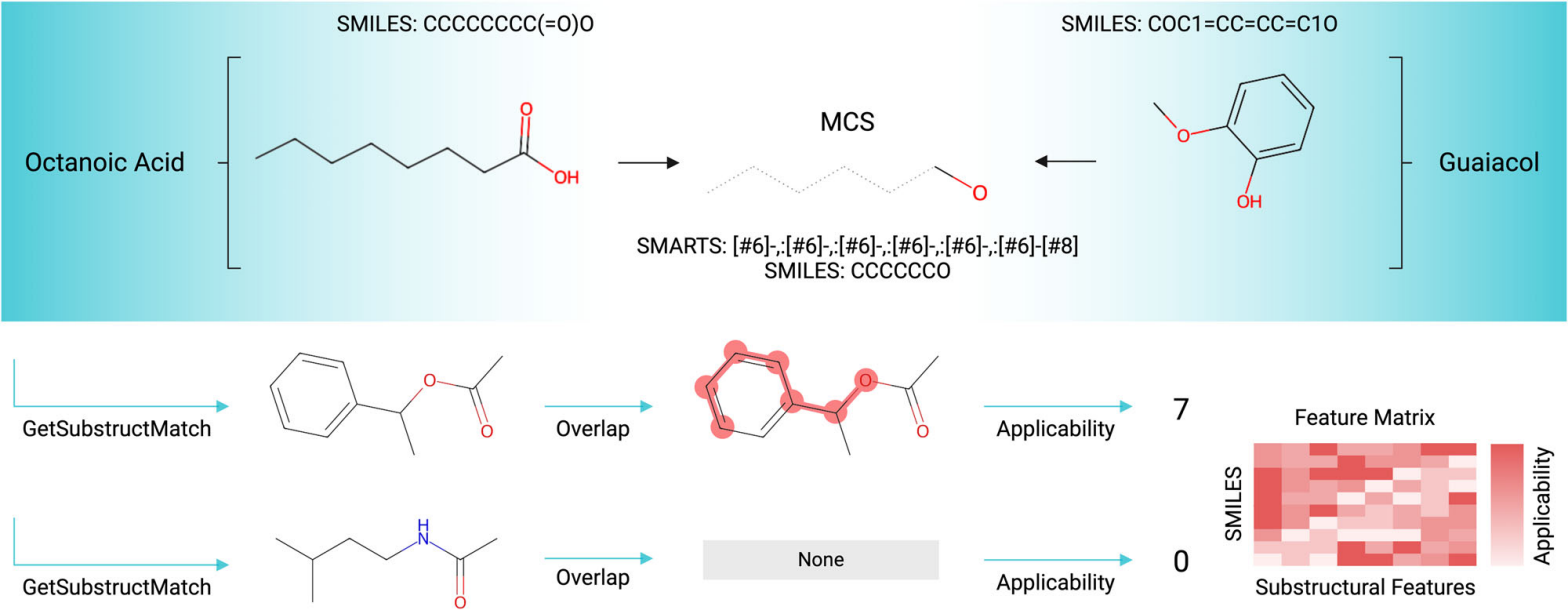
Two example molecules, namely, octanoic acid and guaiacol are shown and the maximum common substructure between the two is calculated using RDKit^57^. The same process is performed for each of the 390 molecules in the reference set. The resulting MCS result is compared to two molecules from the training dataset. The lack of presence of this MCS substructure in the second molecule means that it is assigned an applicability value of zero. This process is then repeated over all MCS substructures, and all molecules detected across each whisky sample to generate the feature applicability matrix shown. Image created with biorender.com.

**Fig. 4:**
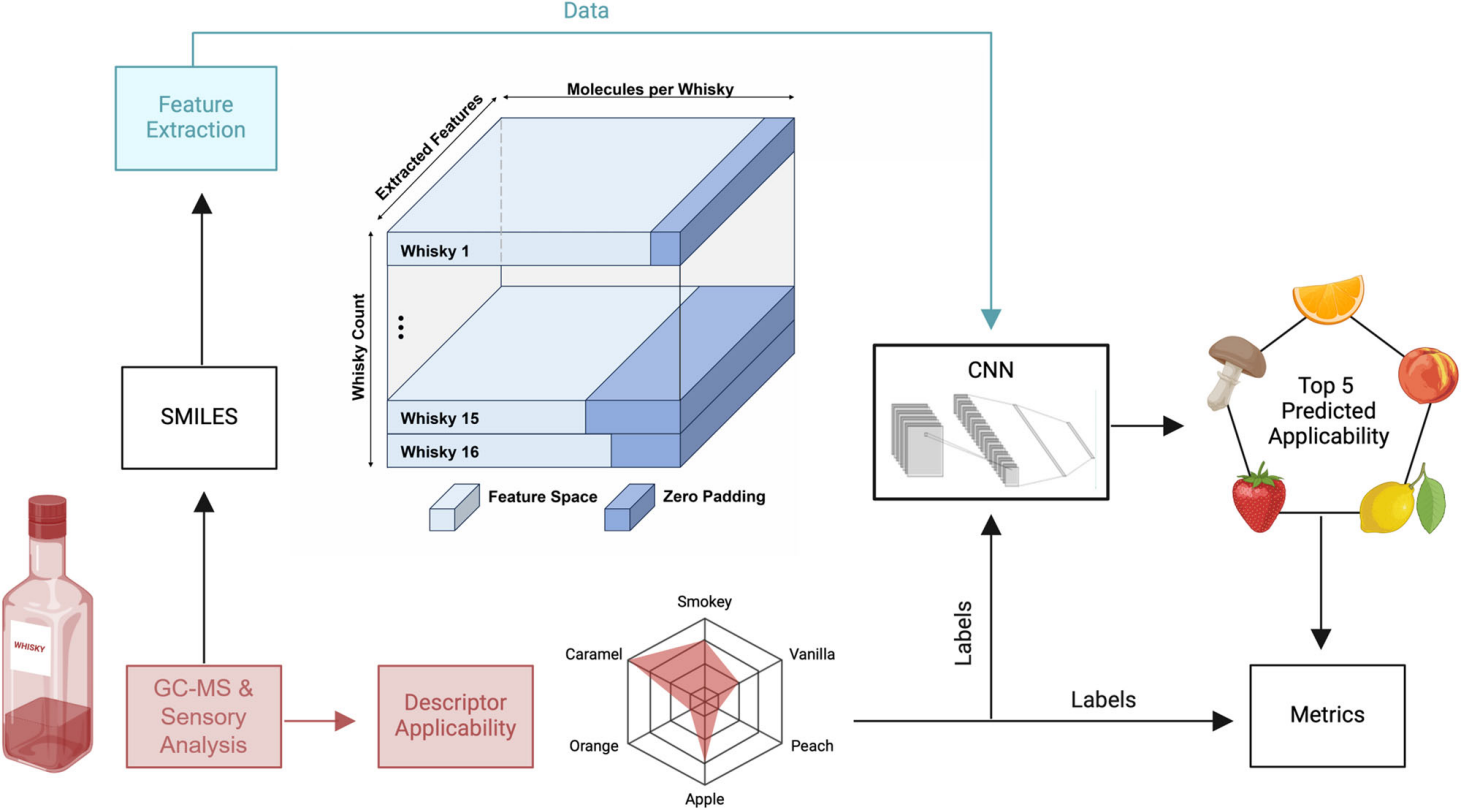
Schematic depiction of the “stack-and-pad” approach for the features extracted per whisky sample for each molecule detected. The whisky samples are analyzed with sensory analytical approaches to identify the applicable descriptors and molecule SMILES. Using the MCS approach, features are extracted using the training and reference dataset. These features are stacked and zero padded to create a feature cube that is passed into the CNN along with the labels for training and the resulting top-5 descriptors are compared to their ground truths. Image created with biorender.com.

These molecules were then compared pairwise to extract the different substructural features by finding the *m*aximum *c*ommon *s*ubstructure (MCS, see Fig. 3) between each of these pairs. Finally, an overlap was calculated between each of the molecules in the training dataset, i.e., all molecules detected across all whisky samples and the MCS results to assign an applicability weight to each of the substructures. These varied from 0 (not relevant) to the length of the overlap (very relevant) and can be considered as the frequency of occurrence of the substructures. The resulting matrix was of the shape (279, 3979) since there were 279 unique molecules detected across all whisky samples and a total of 3979 substructural features were extracted from the reference dataset. An example of this pipeline is shown in Fig. 4. The applicability matrix obtained here was also used for training the CNNs described below.

### Odor descriptor labels

Based on the sensory data evaluation^5^ and on previous work for predicting odor descriptors^22^, we chose to predict the top-5 odor descriptors. To generate labels for predicting the top-5 descriptors per whisky, RATA scores of all panelists were summed and the five descriptors with the highest sum were chosen. In case of a tie, labels were selected randomly. To ensure that the results from OWSum and CNN approaches can be compared, this process was performed once with a fixed seed to ensure that the label selection does not change the top-5 descriptors in cases of ties across different training and evaluation runs. Table 4 provides an overview of the distribution of the descriptors, showing class imbalance. Whereas *flowery* and *honeydew melon-like* are only amongst the top-5 descriptors in two whiskies, while *fruity* occurs in 13 whiskies.

**Table 4:** Distribution of the descriptors across each sample when only the top 5 descriptors are considered shows a class imbalance

Descriptor	Top-5 occurrence
Solvent-like	8
Apple-like	9
Flowery	2
Butter/Butter-Rum-like	3
Fruity	13
Honey-like	5
Caramel-like	6
Peach-like	3
Smoky	4
Phenolic	5
Vanilla-like	3
Pear-like	7
Woody	3
Honeydew melon-like	2
Coconut-like	0
Spicy/Clove-like	3
Orange-like	4

Moreover, the class *coconut-like* was removed for the classification process as it was tied for the 5th place in the top-5 selection and random selection led to the class being dropped, yielding 16 classes, as opposed to the regression where all classes were considered.

### OWSum

OWSum is a linear classification algorithm that is based on statistical measures like conditional probabilities to calculate influence values per feature that further are used to predict one or several classes^21^. OWSum was firstly used to predict the type of whiskies, i.e., Scotch or American whisky. Two types of features were used for this task, on the one hand the detected molecules, on the other hand descriptors identified by the panel for each sample. The labels were binary class labels denoting class Scotch or American whisky. Secondly, we also used OWSum to predict the odor qualities of the whiskies, where, once again, the detected molecules were used as features and the top-5 descriptors as targets. For this case, OWSum predicts the five descriptors that were assigned the highest scores instead of the single highest class.

The respective predictions were performed using either the conditional probability CP1 $\Pr ({{C}}_{i},{{F}}_{j})$ for a feature *F*_*j*_ and a class C_*i*_ or the conditional probability CP2 $\Pr ({{F}}_{j},{{C}}_{i})$ combined with different weighting schemes as described in ref. ^21^.

In the first weighting scheme, no additional weights were used (same-weighted OWSum). In the second case we used tf-idf-weights that were calculated for each LOO iteration based on the training set. The tf-idf value considers the term frequency as well as its specificity and was calculated for each feature per class in the training set according to equation 1. The term frequency is the number of occurrences of a particular feature F_*j*_ within a class C_*i*_ (${{\rm{\#}}}({{F}}_{j},{{C}}_{i})$) divided by the total number of features in this specific class ${len}\left({{C}}_{i}\right)$. The inverse document frequency is the logarithmically scaled division of the number of classes $\left|C\right|$ by the number of classes containing the feature ${\sum}_{{{C}}_{n}:{{\rm{F}}}_{j}\in {{C}}_{n}}1$.

1 $${{tf}-{idf}}_{i,j}=\frac{{{{\#}}}\left({{\rm{F}}}_{j},{{C}}_{i}\right)}{{len}\left({{C}}_{i}\right)}\cdot \log \left(\frac{\left|{{\rm{C}}}\right|}{{\sum}_{{{C}}_{n}:{{\rm{F}}}_{j}\in {{C}}_{n}}1}\right)$$

$${{F}}_{j}$$=feature *j*

$${{C}}_{i}$$=class *i*

$\left|C\right|$=number of classes

$${{C}}_{n}$$=class n.

To account for class imbalance and standardize the dataset on each fold, we used StandardScaler from scikit-learn version 1.2.1. Features that were scaled to positive values were automatically considered as present for this object by OWSum, while those scaled to negative values were considered non-present.

### CNN

The second approach we undertook for predicting odor qualities of whisky samples was to train a CNN on the applicability matrix generated by pairwise comparison of molecules in our dataset. For this purpose, a “stack-and-pad” approach was undertaken since each whisky has a different number of detected molecules, wherein the applicability features for each whisky sample in a batch were stacked together and padded to a common length. For example, whisky 1 could have 195 detected molecules while whisky 2 could have 180 molecules, the applicability features for each of these detected molecules were extracted from the global applicability matrix based on the molecules detected in both samples and stacked together to create a sample specific feature matrix. To ensure that this data could be fed into a 2D-CNN pipeline, further zero padding was performed for each batch. This is shown in Fig. 4. The CNN pipeline consisted of two 2D convolutional layers, an adaptive max pooling layer followed by two fully connected layers to classify the top-5 descriptors. For training, a LOO approach was undertaken where each sample was placed once in the test set and all others were used for training. A binary cross-entropy loss with sample weights was optimized using Optuna^53^ hyperparameter optimization for the ideal training parameters. Additionally, a second weight was introduced for the applicability feature matrix. These are the relative peak areas calculated for each molecule during the analytical assessment of the whisky samples, quantified by their abundance relative to an internal standard 4-chloro-2-methoxyphenol over all molecules detected in each sample^5^.

These relative peak areas were used to scale the input features to serve as a proxy for the concentration or amount of the different substances allowing for different influence of similar compounds found across different whisky samples. Additionally, during the hyperparameter optimization, we allowed the models to also be trained without any feature scaling to observe the change in the evaluation metrics and to compare if the relative peak areas can be in fact used as a proxy for concentration values for both, classification and regression.

Finally, a second weighting/penalty scheme was employed to counter the class imbalance that occurs across the different descriptors for the classification task. As shown in Table 4, upon calculating the top-5 descriptors for each sample, there is a clear class imbalance that can skew the results towards the majority class, i.e., *fruity*, *apple-like,* and *solvent-like* in this case. One approach to counter this, is to use LOO that allows each sample to be treated as a test sample while using the others for training, allowing us to train a model with a small dataset while ensuring that the test results are not due to a favorable data split. Taking inspiration from the Open-POM^54^, we used inverted class weights in the BCE loss while training. These were calculated for each fold independently using Eq. 2, where *fold frequency* is the number of times a descriptor occurs in the train set for each fold and *all frequency* is the overall occurrence of the descriptor in the dataset. These are, however, not to be confused with the weights derived from the relative peak areas that were used as a proxy for concentration or compound amount and used to scale the input features instead of being used with the loss function.

2 $${weights}=\left\{\begin{array}{c}{\hfill 0,} 	 {x=0 \ }	 \hfill \\ {\log \;(1+\frac{y}{x}),} 	 {x \; > \;0,} 	 {\forall x\in {fold\; frequency},{{\rm{y}}}\in {all\; frequency}}\end{array}\right.$$

Similarly, for predicting the raw RATA scores, L1 and MSE loss were chosen and targets were raw RATA scores for each sample. Consequently, the final output from the network was raw RATA scores and not probabilities for top-5 class assignment. For classification, the CNN was trained for 11 epochs and for regression, 21 epochs were used. The other hyperparameters can be found in Table S3.

### SVM and Random Forest

For comparing our methods with other commonly used methods, we used SVM and RF to classify the whisky RATA data. Both methods were trained similar to CNN and OWSum in a LOO approach. For SVM, LinearSVC from sklearn was used as a OneVsRest classifier, i.e., each of the 16 descriptors were considered as a binary classification problem. Moreover, the data in each LOO iteration was scaled using StandardScaler, i.e., fit transformed on the train data and only transformed on the test data to avoid any leakage. Based on the decision scores obtained from each of the SVMs, the top-5 descriptors were chosen as the predictions.

Similarly, RF was also trained in an LOO approach using a similar concept with OneVsRest from sklearn before the five highest probabilities from the RF for class 1 for each descriptor were selected to obtain our predictions. Both of these methods were also optimized using GridSearchCV from sklearn and LOO cross validation. For SVM, 200 maximum iterations were used while RF used 100 estimators.

### Metrics

Initially, to get insight into the data with OWSum, no train-test-split was performed, and all molecules were tested. We report re-creation performances, i.e., the comparison of the true with the predicted type if using the same train and test data and as such re-creating the information.

For other approaches, we performed *l*eave-*o*ne-*o*ut (LOO) cross validation. For the binary classification of the whisky type using OWSum, we calculated raw accuracies (ACC). For the multi-label classifications, we used the micro F1-Score, ROCAUC score (*A*rea *U*nder the *R*eceiver *O*perating *C*haracteristic *C*urve), and micro MCC (*M*atthews *C*orrelation *C*oefficient) and regression results were evaluated with Pearson correlation between the predictions and the ground truth (PCC, *P*earson *C*orrelation *C*oefficient). All metrics were calculated using scikit learn^55^ in Python; due to class imbalance (see Table 4), we used micro metrics. The results from all methods are summarized in Table 2.

### Inter-subject performance (Subject X)

To compare the performance of our methods to that of the experienced panelists, we compared the inter-subject performance also using LOO, i.e., the top-5 descriptors were determined on all-but-one expert, this served as our inter-subjected prediction and the top-5 descriptors of the sum of ratings from all other experts served as test data for comparison and to calculate our metrics. This was repeated for each expert and then averaged. As such, we were able to calculate all metrics we used for our model’s performance measures also for inter-subject performance measures and thus compare algorithm performance to the performance of an average panelist.

### Educated top-5 guessing

The idea behind educated top-5 guessing is to use experiential inference if no information regarding odor descriptors is available for a new whisky sample. One potential method for determining this is through guessing the five most likely descriptors based on established whiskies. As such, we also estimate performance metrics for educated top-5 guessing by first taking the five most frequent descriptors as “educated” guess for each whisky (namely: *solvent-like, apple-like, fruity, caramel,* and *pear-like*) and compare these to the ground truth for all but one whisky in a LOO fashion and repeat for each whisky sample.

## Supplementary information

Supplementary Material

## Data Availability

The source code is openly available at https://osf.io/kyu9r/. Correspondence and data requests should be addressed to andreas.grasskamp@ivv.fraunhofer.de.
