# Supplementary material for: Odor prediction of whiskies based on their molecular composition
Source: Commun Chem. 2024 Dec 19;7:293. doi: 10.1038/s42004-024-01373-2 (PMC11659623; doi:10.1038/s42004-024-01373-2)
Supplement: Supplementary file 1 — Supplementary Material [file 42004_2024_1373_MOESM1_ESM.pdf]

Odor prediction of whiskies based on their molecular composition

Satnam Singh[1,2*], Doris Schicker[1*], Helen Haug[1,3], Tilman Sauerwald[1,4], Andreas T. Grasskamp[1†]

[1]*Department of Sensory Analytics and Technologies, Fraunhofer Institute for Process Engineering and Packaging IVV, Giggenhauser Str. 35, 85354 Freising, Germany*

[2]*Department of Psychiatry and Psychotherapy, Friedrich-Alexander-Universität Erlangen-Nürnberg, Schwabachanlage 6, 91054 Erlangen, Germany*

[3]*Department of Chemistry and Pharmacy, Chair of Aroma and Smell Research, Friedrich-Alexander-Universität Erlangen-Nürnberg, Henkestraße 9, 91054 Erlangen, Germany*

[4]*Department of Systems Engineering, Saarland University, Campus A5 1, 66123 Saarbrücken, Germany*

*These authors contributed equally

[†]Correspondence should be addressed to andreas.grasskamp@ivv.fraunhofer.de

– Supplementary material –

1. Predicting the type of whisky with OWSum

Table S1: Accuracy for LOO to predict the type of whisky (American or Scotch) using OWSum

Model	Features	ACC [%]	Not predictable
OWSum (CP1, same-weighted)	Descriptors	93.75	0
OWSum (CP1, tf-idf-weighted)	Descriptors	75.00	0.06
OWSum (CP2, same-weighted)	Descriptors	81.25	0
OWSum (CP2, tf-idf-weighted)	Descriptors	75.00	0.06
OWSum (CP1, same-weighted)	Molecules	93.75	0
OWSum (CP1, tf-idf-weighted)	Molecules	100	0
OWSum (CP2, same-weighted)	Molecules	81.25	0
OWSum (CP2, tf-idf-weighted)	Molecules	100	0

2. Predicting the odor quality of whiskies with OWSum and CNN

Table S2: Evaluation metrics for each of the LOO iterations for CNN, OWSum, Subject X and the benchmarking methods SVM and RF results

**Metric**	**mean**	**std**	**min**	**25%**	**50%**	**75%**	**max**
**CNN (no scaling)**							
**F1**	0.66	0.19	0.33	0.57	0.57	0.89	0.89
**MCC**	0.65	0.17	0.38	0.56	0.56	0.86	0.86
**ROCAUC**	0.76	0.12	0.60	0.70	0.70	0.90	0.90
**OWSum (CP2, tf-idf-weighted)**							
**F1**	0.61	0.15	0.40	0.60	0.60	0.60	1.00
**MCC**	0.44	0.22	0.13	0.42	0.42	0.42	1.00
**ROCAUC**	0.72	0.11	0.56	0.71	0.71	0.71	1.00
**Subject X**							
**F1**	0.35	0.13	0.14	0.24	0.39	0.46	0.54
**MCC**	0.15	0.18	-0.15	0.00	0.19	0.28	0.44
**ROCAUC**	0.57	0.08	0.43	0.49	0.58	0.64	0.69
**Top-5 guessing**							
**F1**	0.52	0.21	0.20	0.40	0.40	0.65	0.80
**MCC**	0.29	0.30	-0.16	0.13	0.13	0.49	0.71
**ROCAUC**	0.65	0.15	0.42	0.56	0.56	0.75	0.85
**SVM**							
**F1**	0.59	0.19	0.20	0.55	0.60	0.60	1.00
**MCC**	0.40	0.27	-0.16	0.35	0.42	0.42	1.00
**ROCAUC**	0.70	0.14	0.42	0.67	0.71	0.71	1.00
**Random Forest**							
**F1**	0.61	0.17	0.40	0.40	0.60	0.80	0.80
**MCC**	0.44	0.25	0.13	0.13	0.42	0.71	0.71
**ROCAUC**	0.72	0.12	0.56	0.56	0.71	0.85	0.85
**CNN (without scaling)**							
**PCC**	0.68	0.15	0.37	0.59	0.70	0.76	0.89

Figure S3: Comparison of panelist performance against OWSum and CNN for each sample. No one panelist consistently outperforms or matches the performance of the algorithm. However, the samples of type Scotch led to a higher disagreement amongst the panelists compared to American whisky and also seems more challenging for the algorithms to train. Our algorithms, however, consistently outperform the mean of the subjects per whisky.

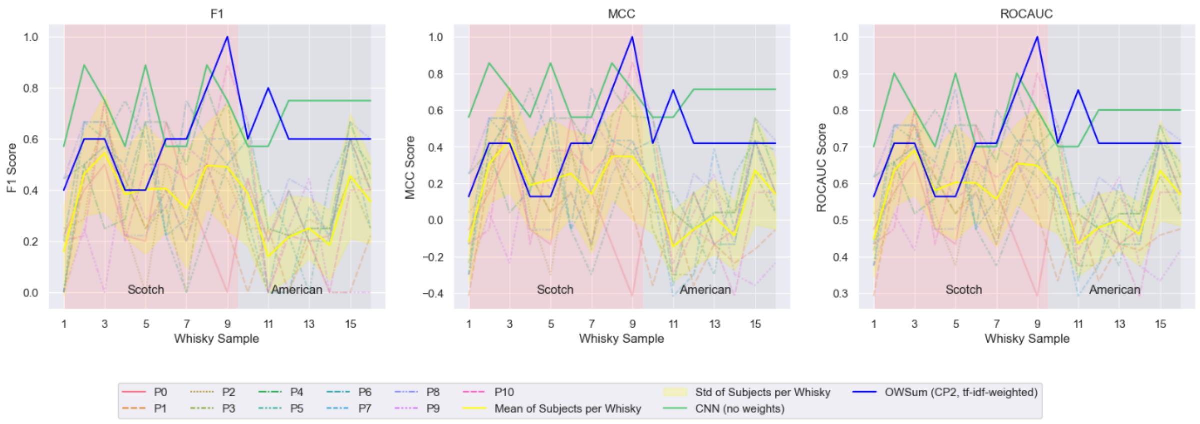

Table S3: Hyperparameters for different CNN models are provided here for reproducibility

Hyperparameter	*CNN (Classification)*	*CNN (Regression)*
Epochs	11	21
Learning Rate	0.0099016	0.0086539
Weight Decay	6.8291298e-06	1.1509616e-07
Batch Size	3	2
Feature Size	6	7
Rate Decay Epoch	12	-

3. Importance of top molecules per descriptor

Table S4: Molecules with the two highest importance values per descriptor based on tf-idf-weighted CP2 OWSum, re-creation accuracy: 96.88 %. Aroma descriptors and the corresponding importance values should not imply causal relationships between molecule and odor quality.

Molecule	Importance value
Solvent-like	
(4*R*)-1-methyl-4-prop-1-en-2-ylcyclohexene	0.001337
cumene	0.001337
hex-1-en-3-one	0.001337
1-methoxynaphthalene	0.001586
3-chloro-4-propan-2-yloxybenzonitrile	0.001586
heptan-3-one	0.001586
Apple-like	
methyl octanoate	0.001276
(3*R*)-3-hydroxy-4,4-dimethyloxolan-2-one	0.001412
4-chlorophenol	0.001412
propanoic acid	0.001412
Flowery	
methyl octanoate	0.001997
(1*E*,4*E*,8*E*)-2,6,6,9-tetramethylcycloundeca-1,4,8-triene	0.002513
(2*E*,4*E*)-nona-2,4-dienal	0.002513
(methyltrisulfanyl)methane	0.002513
1,2-xylene	0.002513
1,3-xylene	0.002513
1,5,9-trimethyl-12-propan-2-yl-15-oxabicyclo[10.2.1]pentadeca-5,9-dien-2-ol	0.002513
2-pentyl-2,3-dihydropyran-6-one	0.002513
2,6-dimethoxyphenol	0.002513
2,6-dimethyloct-7-en-2-ol	0.002513
4-hydroxy-2,5-dimethylfuran-3-one	0.002513
4,4,7a-trimethyl-6,7-dihydro-5H-1-benzofuran-2-one	0.002513
5-heptyloxolan-2-one	0.002513
Butter/Butter-Rum-like	
(2*E*,4*E*)-hepta-2,4-dienal	0.001844
4,4,7a-trimethyl-6,7-dihydro-5*H*-1-benzofuran-2-one	0.001844
4-propan-2-ylbenzaldehyde	0.001844
4-methylhexanoic acid	0.001844
4-hydroxy-2,5-dimethylfuran-3-one	0.001844
2,6-dimethoxyphenol	0.001844
2-pentyl-2,3-dihydropyran-6-one	0.001844
butan-2-ylbenzene	0.001844
2-hydroxybenzaldehyde	0.001844
1,3-xylene	0.001844
1,2-xylene	0.001844
1-chloro-2-methoxybenzene	0.001844
(methyltrisulfanyl)methane	0.001844
(2*E*,6*E*)-nona-2,6-dienal	0.001844
(2*E*,4*E*)-nona-2,4-dienal	0.001844
2-hydroxy-3-methylcyclopent-2-en-1-one	0.001844
methyl prop-2-enoate	0.001844
2-phenylacetaldehyde	0.002197
Fruity	
(*E*)-4-(2,6,6-trimethylcyclohexen-1-yl)but-3-en-2-one	0.000995
1,2-oxazole	0.000995
2-ethylhexyl prop-2-enoate	0.000995
2-methylbutan-1-ol	0.00111
Honey-like	
3,3-dimethylbutanoic acid	0.00137
8-tricyclo[5.2.1.02,6]dec-3-enyl acetate	0.00137
2-octyloxirane	0.00189
3-methylbut-2-en-1-ol	0.00189
Caramel-like	
2-octyloxirane	0.001548
(*E*)-2-ethylhex-2-enal	0.001754

Molecule	Importance value
Peach-like	
1-chloro-2-methoxybenzene	0.002037
2-methoxy-4-[(*E*)-prop-1-enyl]phenol	0.002037
4-propan-2-ylbenzaldehyde	0.002037
5-methylhexanoic acid	0.002037
butan-2-ylbenzene	0.002037
hexanoic acid	0.002037
methyl prop-2-enoate	0.002037
oxolane-2,5-dione	0.002037
pentanoic acid	0.003641
Smoky	
(*E*)-3-phenylprop-2-enal	0.00314
1-(4-methoxyphenyl)propan-2-one	0.00314
1-(4-methylphenyl)ethanone	0.00314
2-propan-2-ylphenol	0.00314
3-ethylphenol	0.00314
4-propan-2-ylphenol	0.00314
4-propylphenol	0.00314
2,4-dimethylphenol	0.003511
Phenolic	
(*E*)-3-phenylprop-2-enal	0.002762
1-(4-methoxyphenyl)propan-2-one	0.002762
1-(4-methylphenyl)ethanone	0.002762
2-propan-2-ylphenol	0.002762
3-ethylphenol	0.002762
4-propan-2-ylphenol	0.002762
4-propylphenol	0.002762
2,4-dimethylphenol	0.003087
Vanilla-like	
heptan-1-ol	0.000958
(2*E*,4*E*)-hepta-2,4-dienal	0.002372
(2*E*,6*E*)-nona-2,6-dienal	0.002372
2-hydroxy-3-methylcyclopent-2-en-1-one	0.002372
2-hydroxybenzaldehyde	0.002372
4-methylhexanoic acid	0.002372
[(1*R*,2*R*,4*R*)-1,7,7-trimethyl-2-bicyclo[2.2.1]heptanyl] prop-2-enoate	0.002372

Molecule	Importance value
Pear-like	
(4*R*)-1-methyl-4-prop-1-en-2-ylcyclohexene	0.001549
cumene	0.001549
hex-1-en-3-one	0.001549
pentanoic acid	0.001642
Woody	
1-methoxynaphthalene	0.003687
3-chloro-4-propan-2-yloxybenzonitrile	0.003687
heptan-3-one	0.003687
(*E*)-3-phenylprop-2-enal	0.003931
1-(4-methoxyphenyl)propan-2-one	0.003931
1-(4-methylphenyl)ethanone	0.003931
2-propan-2-ylphenol	0.003931
3-ethylphenol	0.003931
4-propan-2-ylphenol	0.003931
4-propylphenol	0.003931
Honeydew melon-like	
6-butyloxan-2-one	0.001038
ethyl (*Z*)-octadec-9-enoate	0.001038
ethyl 2-methylpentanoate	0.001038
2-methoxy-4-[(*E*)-prop-1-enyl]phenol	0.003484
prop-2-enoic acid	0.003484
Spicy/Clove-like	
(1*R*,2*R*,4*R*)-1,2,7,7-tetramethylbicyclo[2.2.1]heptan-2-ol	0.001928
7-methyl-3-methylideneocta-1.6-diene	0.001928
5-methyl-2-propan-2-ylphenol	0.001928
4-pentylphenol	0.001928
4-hexylphenol	0.001928
3-sulfanylbutan-2-one	0.001928
2,5-dichlorophenol	0.001928
2,3,5-trimethylpyrazine	0.001928
[(1*R*,2*R*,4*R*)-1,7,7-trimethyl-2-bicyclo[2.2.1]heptanyl] prop-2-enoate	0.001928
2,3,5-trimethylphenol	0.001928
2-ethoxyphenol	0.001928
1,3-benzothiazole	0.001928
1-fluoro-2-methoxybenzene	0.001928
1-(2-methylphenyl)ethanone	0.001928
(4-methylphenyl) benzoate	0.001928
(3-acetylphenyl) acetate	0.001928

Molecule	Importance value	Molecule	Importance value
(1*S*,5*R*)-4-methyl-1-propan-2-ylbicyclo[3.1.0]hexan-3-one	0.001928		
2-ethyl-3,5-dimethylpyrazine	0.001928	Orange-like	
1-methoxynaphthalene	0.003856	(2*E*,6*Z*)-nona-2,6-dien-1-ol	0.001656
3-chloro-4-propan-2-yloxybenzonitrile	0.003856	(5*S*)-2-methyl-5-prop-1-en-2-ylcyclohex-2-en-1-one	0.001656
heptan-3-one	0.003856	2-sulfanylpropanoic acid	0.001656
		6-methylhept-5-en-2-one	0.001656
		[(1*R*,2*R*,4*R*)-1,7,7-trimethyl-2-bicyclo[2.2.1]heptanyl] prop-2-enoate	0.001656
		benzyl 2-bromoacetate	0.001656
		dimethyl pentanedioate	0.001656
		ethyl (*Z*)-hexadec-9-enoate	0.001656
		ethyl 2-sulfanylpropanoate	0.001656
		methyl undecanoate	0.001656
		N,*N*-dimethylmethanamine;hydrochloride	0.001656
		(4*S*)-4-prop-1-en-2-ylcyclohexene-1-carbaldehyde	0.001974
		1-ethoxypropan-2-yl acetate	0.001974

The influence, however, should be interpreted in relative terms between the classes: for example, the molecule heptan-1-ol has the second highest influence value within the class vanilla-like with 0.000958. However, this molecule has even a higher influence values for the class caramel-like with 0.000990. This is not visible in the table, as the influence for caramel-like is not within the two highest influence values for caramel-like. Moreover, it is also to note that the aroma of the individual molecules with the highest influence might not be the same as the aroma of the mixture.
